# Children can rate perceived effort but do not follow intensity instructions during soccer training

**DOI:** 10.3389/fspor.2023.1251585

**Published:** 2023-11-02

**Authors:** Marco Reinke, Gerd Schmitz

**Affiliations:** Leibniz University Hannover, Institute of Sports Science, Hannover, Germany

**Keywords:** perceived effort, perceived exertion, RPE, executive functions, selfregulation, children, soccer

## Abstract

The perception of effort is elementary for the self-regulation of exercise intensity in sports. The competence for rating perceived effort (RPE) seems to be related to physical and cognitive development. Children accurately rate perceived effort during incremental exercise tests when loads progressively increase, but it remains unclear how children perform when they participate in sports games, which are characterized by complex tasks with varying intensity profiles. The present study investigates children's competencies for rating perceived effort and producing predetermined intensities during soccer training. Twenty-five children aged 11–13 years performed two similar training sessions. In the first session, the children trained without intensity instructions and continuously rated their effort. In the second session, the children were instructed to produce predefined intensities. Before the first training session, executive functions were assessed by cognitive performance tests and a self-report measure. RPE correlated significantly with heart rate measures (*R*^2^^ ^= 0.27, *p* < 0.001). As confirmed by factor analysis, individual differences in these correlations were related to the outcomes of the cognitive tests and the self-report measure. RPE in training session 2 differed from RPE in training session 1 (*d* = 1.22, *p* < 0.001), although the heart rate data did not differ significantly between training sessions (*d* = −0.19, *p* = 0.780). Thirteen-year-old children performed significantly better than eleven-year-old children (*d* = 1.69, *p* = 0.027). The results suggest that children are able to rate perceived effort during soccer training and that this ability is related to executive functions. Conversely, children may not be able to alter their intensities in response to instructions, although their ratings suggest that they have largely succeeded in doing so.

## 1. Introduction

The majority of sports activities are performed without a trainer or teacher supervising the performance. Accordingly, athletes regulate movement intensities autonomously. The principle component of goal-directed intensity regulation in sports is considered to be an adequate subjective perception of effort (1, 2). The competence for rating perceived effort (RPE) is typically evaluated by correlating RPE with objective measures such as heart rate (HR) or oxygen uptake. It is assumed that the RPE-competence improves with age. For example, Rice et al. (3) observed lower correlations between RPE and heart rate in six- to eight- than in eleven- to twelve-year-old children. Yelling et al. (4) described lower correlations in eleven- and twelve- compared to fourteen- and fifteen-year-olds. Conversely, other studies found large and excellent correlations between RPE and HR or performance measures already in nine- and twelve-year-olds (5, 6). Thus, several factors appear to influence how well children can rate perceived effort. One factor is the applied rating scale. For example, Lamb et al. (5) reported higher correlations between RPE and HR in children when effort was rated with a child-specific rating scale compared to the Borg scale, which is the standard scale used for adults. Child-specific scales should include numerical and pictorial representations of effort to support understanding (7). Other factors discussed are the exercise type as well as the familiarity with the scale, the task and intensity ranges (7–9). Finally, RPE-competence depends on cognitive development which may explain differences between age groups as well as the advantages of child-specific rating scales (3, 9, 10).

Relationships between RPE competence and cognitive abilities have been reported for adults. Arnhold et al. (11) found that people with intellectual disabilities (ID) show lower correlations between HR and RPE during incremental exercise tests than people without ID. Schmitz et al. (12) reported similar findings in a field study on soccer training. As a sports game, soccer is characterized by complex tasks with open outcomes and temporally varying intensity profiles. The authors found that the HR measured at the time when effort was rated (current heart rate—cHR) explained one portion of the RPE variance thereby indicating that the current energetic effort contributes to RPE (13). Another portion of the RPE variance was explained by the cumulated training time, suggesting that also cumulative intensity effects contribute to RPE. Differences between people with ID and without ID were evident regarding the variance component explained by cHR. They were partially explained by performance differences in tests measuring the speed of cognitive information processing and flexibility, which are components of the executive control system (14). Schmitz and Sommer (15) amended these findings in another study on soccer training. RPE-variance explained by cHR was again related to the speed of information processing as well as to self-report measures of executive functions. RPE-variance explained by cumulated training effects, which were assessed with Edwards' summated heart rate zone method (sHRz) (16), was not related to performance in the cognitive tests. In summary, these studies suggest a relationship between executive control and the component of RPE explained by cHR but not the component explained by sHRz. Whether similar relationships exist in children is unclear. Since children's executive functions are not yet fully developed, this may explain why some studies found differences between the RPE-competences of different age groups [see also (3, 10)]. The present study investigates the hypothesis of a relationship between executive control and the variance component of RPE explained by cHR in children. The study applies the estimation paradigm from Schmitz and Sommer in children performing soccer training (15). It has been shown for adults, that cHR and sHRz explain independent variance components of RPE (15). Correlations between cHR and RPE are also evident in children [*e.g.* (3, 5, 6),]. sHRz correlates with session RPE which is a subjective measure for the training load of a sport activity. Medium correlations have been reported for eleven-year-old children (*r* = 0.34) playing soccer. Medium to high correlations have been shown for twelve- to seventeen-year-old children and adolescents (r ranging from 0.48 to 0.90) playing soccer or performing other sport-activities (17–22).

The first part of the present study focuses on the estimation of intensities whereas the second part investigates self-regulation of exercise intensities. Self-regulation of exercise intensities is typically investigated with a production paradigm, in which participants produce externally instructed intensities (23). Although this form of self-regulation builds on the perception of effort, it addresses further skills (24). For example, the perception of effort is based on afferent feedback as well as feedforward anticipation of imminent movement effects, whereas intensity regulation requires the prediction of future exertion states on a time scale from minutes or hours. This prediction is continuously compared with the intended state, on the basis of which motor output is adjusted (23, 25).

Studies have shown that children are capable of producing externally instructed intensities. Previously, the majority of studies applied incremental exercise tests (23) with loads that increase continuously. The predictability of future loads may simplify the rating of perceived effort and intensity regulation. Conversely, when children perform sports in a natural environment, their activities are characterized by changing intensity requirements. It is plausible to assume that the natural environment further increases the demands on self-regulation. However, few studies have investigated the production competence with randomly varying intensities. Children aged seven to eleven years were able to consistently produce three randomly varying intensity levels during cycle ergometry and stepping (26) thereby indicating that children can also meet this challenge. Lamb (5) stated that production in a controlled laboratory setting might be performed differently compared to production in an external valid setting like a physical education class. Accordingly, children aged six to eleven years seem to perform worse during a physical education class. In a study from Cowden and Plowman (27), 38% percent of children were not able to regulate exercise intensities within a defined HR range from 130 to 180 beats per minute. 60% showed inconsistent performance over time. These results suggest that these children may not have had the ability to estimate or produce intensities during a physical education class. As such, whether children have the ability to produce intensities in sporting situations with varying intensities remains unknown. The second part of the present study tested the hypothesis that children are able to produce prescribed intensities while playing soccer (production paradigm). Since some results from previous studies suggest an influence of age on the estimation, as well as production competence, the present study aimed to determine if the performances in the estimation and the production paradigm depend on age (10).

## 2. Methods

Twenty-five male children participated in the present study. Mean age was 12.0 years (standard deviation SD: 0.9 years) and all children were free from overt orthopedic or psychic impairments. All participants were members of a regional soccer club and had completed at least 180 min of soccer training per week for the previous four weeks prior to the start of the study. The participants and their legal representatives gave their written informed consent to participate in the study. The study was performed in accordance with the Declaration of Helsinki and had been pre-approved by the Ethics Committee of the Leibniz University Hannover (ID 209_10_2021).

### 2.1. Procedure

The participants first performed cognitive tests and then participated in two training sessions separated by one week. A flowchart on the study design is available as Supplementary File (Flowchart).

#### 2.1.1. Soccer training

The children performed two 90-minute training sessions. All children played in a group with children of the same age only (11 years: *n* = 9, 12 years: *n* = 7, 13 years: *n* = 9). The training sessions were identical for all age groups. They were supervised by the same trainers, who in turn instructed the same exercises with the same settings in both training sessions. The sessions comprised of 10 tasks: Running exercises (task 1); passing, running, and scoring (task 2); passing in dynamic environments (task 3); possession play against an outnumbered team (tasks 4–6); dribbling and scoring without and with time pressure (tasks 7 & 8); free play (tasks 9 & 10).

#### 2.1.2. Heart rate and RPE-competence

Heart rate was measured with a team system (acentas GmbH, Hoegertshausen, Germany, frequency 1 Hz). The participants wore a breast belt, which transmitted data wirelessly to a laptop computer. Markers were set to the data denoting the time-points when perceived effort was rated. Maximum HR was calculated according to a formula from Hottenrott et al. (28), which had been derived from a regression of maximum heart rates (HRmax) and RPE measures from 1,600 participants aged 10 to 70 years (intercept: 207.7, slope: 0.64). Applied to the present sample, the formula resulted in a calculated HRmax of 200.00 (SD: 0.56). If a higher HR was measured during training, the calculated value was substituted with the measured value. The individual HR data were assigned to HR zones: zone 1: ≤60%; zone 2: >60 and ≤70%; zone 3: >70 and ≤80%; zone 4: >80 and ≤90%; and zone 5: >90% of the individual HRmax). The training load of each task was evaluated with the summated HR zone method described by Edwards (16), which yields a summated HR zone score (sHRz). The durations spent in the HR zones are multiplied with the weighting factors 1–5 and then summated; that is, the duration spent in zone 1 is multiplied with the weighting factor 1 and the duration spent in zone 5 is multiplied with the weighting factor 5.

Perceived effort was rated with a modified OMNI Scale (29, 30) which is one of several scales recommended for children (31). The wording of the original OMNI Scale had been adapted for the present study: In order to focus on the rating of effort, the lowest scale value was represented by the term “gar nicht anstrengend”, which is the German translation of the terms “no effort at all” and “no exertion at all”. Different degrees of effort were differentiated with the terms “leicht” (translation of “easy” “light”), “mittel” (“intermediate”), “schwer” (“hard”/“heavy”) and “sehr sehr schwer” (“very very hard/heavy”). These were anchor terms of a scale in a study from Baschta and Lange (32), in which 12- to 14-year-old participants were able to differentiate different degrees of exertion during a 6-min-run. For the rating, the investigators called each player to the sideline, asked for the effort, and instructed him to indicate the perceived effort by pointing on the OMNI Scale. Perceived effort was rated at the end of each training task, on average every 9.2 (SD: 1.3) minutes.

### 2.2. Production of intensities

All participants performed a second training session with the same contents and durations as the first session. For one participant, RPE of the second session was not recorded due to an error in documentation. Therefore, in the second training session, only his HR data were analyzed. Before each exercise started, each participant was informed about his RPE in the same task of the first session and then instructed to train with a pre-determined intensity slightly below, equal or higher than before: 64% of the effort ratings during the first session were lower than the value 5 on the OMNI-scale, which might indicate that the children predominantly exercised below the ventilatory threshold (33). In these cases, it did not seem reasonable to instruct lower intensities. Therefore, on average, each child was instructed at a higher intensity in training session 2 than in training session 1 (mean difference of OMNI-scale values: 1.76, SD: 0.48, range: −2–+4). At the end of each training task, each participant again rated the perceived effort.

### 2.3. Cognitive assessment

Two non-verbal neuropsychological tests (paper-pencil-tests) and one questionnaire were applied. The tests used were the same tests that proved correlations between cognitive performance factors and the perception of effort in adults and adolescents in previous studies (12, 15, 34).

The Number-Connection-Test measures the speed of information processing. Within 30s, the participants have to connect on a DIN A4 sheet of paper numbers in ascending order as quickly as possible. Consecutive numbers are located directly above, below, to the left, to the right, or at a diagonal position to each other. Performance time is transformed into a measure that informs about how many Bits can be cognitively processed within one second (Bit/s). The outcome is related to fluid intelligence and correlates with medium-to-large effects with the outcome of intelligence tests (35, 36). Furthermore, information processing is one of four core areas within a framework on executive control described by Anderson (14) from a developmental perspective.

The Five-Point-Test provides measures for spontaneous and reactive flexibility. Within 3 min, the participants have to produce as many unique designs as possible while avoiding repetitions. A design is produced by connecting two to five dots, which are preprinted in a rectangle. A sheet of paper (DIN A4) contains forty rectangles, and the participants worked on one to two sheets of paper depending on their performance speed. The present study applied the HAMASH-version published by Haid et al. (37). The number of unique designs reflects spontaneous flexibility; the number of repeated designs reflects cognitive flexibility emerging from inhibition, i.e., reactive flexibility (38, 39). Both measures are compared with normative data of 11–14 year old children (40, 41). Fluency is also considered to be part of the domain of information processing within the framework of Anderson (14) on executive control.

Furthermore, the participants provided a self-report on executive functions by answering the BRIEF-questionnaire (Behavior Rating of Executive Functioning for children). In the questionnaire, they were asked about the occurrence of various behavioral aspects in everyday life during the past months. From the responses, a behavioral regulation index was calculated as a measure for the self-view on the “inhibition of thoughts and actions, flexibility in shifting the problem-solving set, regulation of emotional responses, and monitoring one's actions” (42), p. 23). A German version of the questionnaire and normative data are provided by Drechsler and Steinhausen (43).

### 2.4. Statistics

The assumption of normality was tested with the Shapiro–Wilk-Test and the assumption of variance homogeneity was tested using the Levene's test. Any violations to the assumptions of normality or homogeneity are mentioned specifically in the results section.

The results of the Number-Connection-Test and the BRIEF-questionnaire were compared with data from normative samples using a one-sample *t*-test (36, 43). The data from the Five Point Test was compared with data from thirty 11–14 year old participants provided by Risser and Andrikopoulos (40) published in Spreen & Strauss (41) by an independent samples *t*-test (Section 3.1).

The intensity distributions were analyzed in Section 3.2 by a one-way ANOVA with the within-subject factor heart rate zone. Due to the significance of Mauchly's test, which indicates a violation of the sphericity assumption, the *p*-level was corrected according to the Huyhn-Feldt-procedure. Post-hoc comparisons were performed using a Tukey's post-hoc test.

Relationships between RPE, cHR, and sHRz were analyzed in Section 3.3 with a hierarchical linear model (HLM) with “participant” as a random level 2 predictor and cHR as well as sHRz as fixed level 1 predictors. The restricted maximum likelihood method was chosen as the estimation method and the degrees of freedom were calculated with the Kenward-Roger-Approximation. It was controlled that the Akaike information criterion (AIC) of each HLM was higher than the AIC of a null model with “participant” as a single predictor. Partial correlations between RPE and cHR as well as RPE and sHRz were calculated for each participant by individual regression analyses. Z-transformed semi-partial correlation coefficients were used in the following analyses (44). The regression equations from the individual regression analyses allowed calculations of prediction values for RPE from cHR and sHRz in training session 2.

The hypothesis that the RPE-variance explained by cHR is related to the results of the cognitive performance tests and the BRIEF-questionnaire was tested with confirmatory factor analysis. In the confirmatory factor analysis, this hypothesis is the global null hypothesis. Because the model is confirmed by an insignificant result, it is reasonable to assess model validity with further descriptive measures. According to Hu and Bentler (45), the following indices and suggested cut-off values were defined: root mean square error of approximation (RMSEA, ≤0.06), comparative fit index (CFI, ≥0.95), Tucker Lewis Index (TLI, ≥0.95) and standardized root mean square residuals (SRM, ≤0.08). Furthermore, the hypothesis is confirmed if the factor loadings of each cognitive variable as well as of cHR are significant. If sHRz is added to the model, its factor loadings should not be significant.

The data from training session 2 were analyzed in Section 3.4 using repeated-measures ANOVAs. One ANOVA was performed with the within-subject factor RPE measure (rating session 1, instruction session 2, prediction session 2, rating session 2). Two separate ANOVAs were calculated for cHR and sHRz with the within-subject-factor session (1 vs. 2).

In Section 3.5, age group effects for the estimation competence were analyzed using a one-way ANOVA with the between-subject factor age and the dependent variable factor score, which was derived from the above-described confirmatory factor analysis. Age group effects for the production competence were analyzed by entering age as a between-subject factor to the respective ANOVAs.

## 3. Results

### 3.1. Cognitive performance and self-report

According to the results of the number connection test, the children were able to cognitively process on average 1.83 Bits per second (SD: 0.55, 95% CI: 0.45). The children performed about half a standard deviation worse (mean z: −0.55, SD: 1.22, 95% CI: 1.01) compared to the mean of the normative sample [mean z: 0, SD: 1, *t*(24) = −2.24, *p* = 0.035, *d* = −0.45]. In the Five-Point Test, the participants produced 32.36 (SD: 7.67, 95% CI: 6.33) unique designs. Compared to normative data (mean 29.5, SD: 7.77, *n* = 30) of 11–14 year old participants, the performance of the present sample was not significantly different [*t*(53) = 1.37, *p* = 0.177, *d* = 0.37]. The participants produced 1.80 (SD: 1.71, 95% CI: 1.41) perseverations. Again, there was not a significant difference compared to normative data [mean: 1.27, SD: 1.76, *t*(53) = 1.13, *p* = 0.265, *d* = 0.31]. The mean behavioral regulation index from the BRIEF questionnaire was 63.48 (SD: 7.82, 95% CI: 6.46). Compared with data from the normative sample, the participants of the present study rated their behavioral control during daily activities lower than age-matched controls [*t*(24) = 2.42, *p* = 0.023, *d* = 0.48].

### 3.2. Exercise intensities in training session 1

The children reached an average HRpeak of 192.96 (SD: 8.99, 95% CI: 7.26) beats per minute, which was significantly lower than the calculated HRmax of 200.00 [SD: 0.56, 95% CI: 0.45, *t*(25) = −4.05, *p* < 0.001, *d* = −0.79]. The HRpeak of six children (mean: 205.17, SD: 4.31, 95% CI: 9.04) was higher than the calculated HRmax. Therefore, HRpeak was used to calculate their heart rate zones.

**Figure 1:**
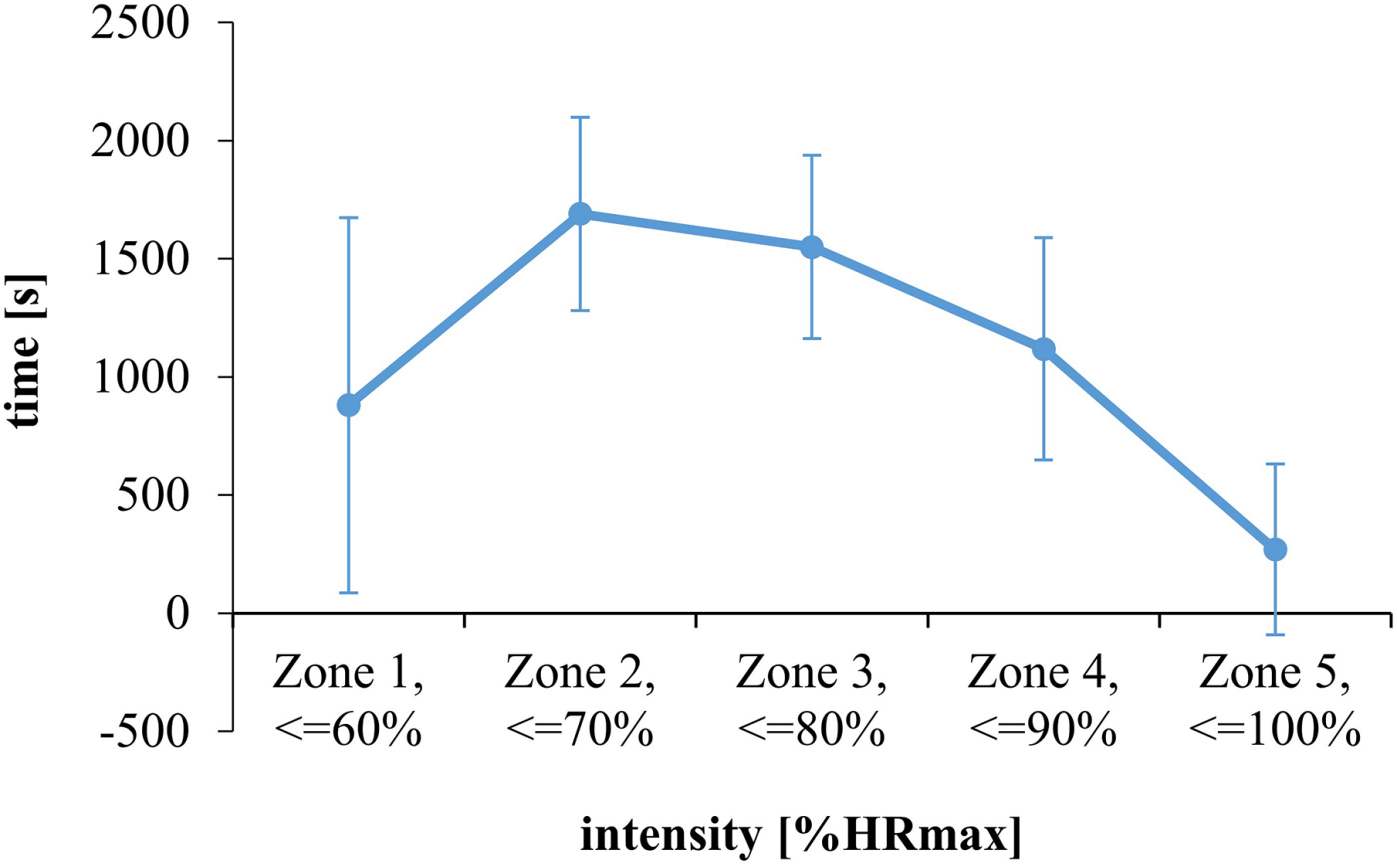
Training volume at five different heart rate zones during training session 1 (estimation task). Data are illustrated as inter-individual means and standard deviations.

Figure 1 illustrates the temporal distribution of the training in relation to the heart rate zones in training session 1. The children trained for the largest amount of time with submaximal intensities between 60% and 80% of the HRmax. The participants trained significantly longer in zones 2 and 3 than in the other zones [*F*(4.96) = 24.90, *p* < 0.001, *ɳ*_p_^2 ^= 0.51, post-hoc test: at least *p* < 0.05]. The least amount of time was trained with more than 90% of HRmax (all *p* < 0.05). The time trained at the highest intensity was still significantly different from zero [*t*(24) = 3.72, *p* = 0.001, *d* = 0.74].

### 3.3. RPE-competencies in training session 1

**Figure 2:**
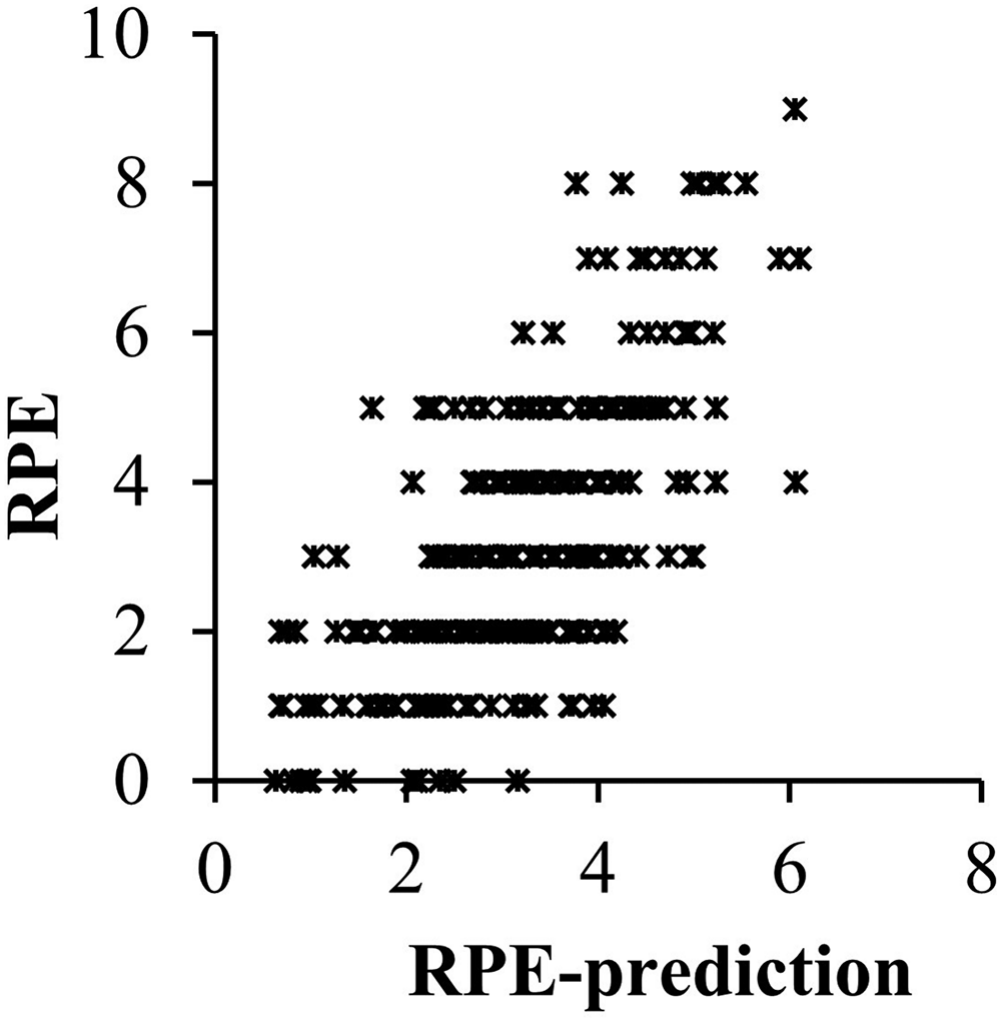
Plots of RPE and RPE-prediction from the hierarchical linear model (HLM) with current heart rate and the summated heart rate zone score in training session 1 as level 1 predictors and participant as level 2 predictor.

The children rated perceived effort 10 times every 9.2 min (SD: 1.3). At the time of a rating, the cHR was measured. The sHRz was calculated for the time interval that had passed since the last rating. To analyze whether the ratings of perceived effort are related to the heart rate data, a hierarchical linear model was calculated with RPE as the criterion variable and cHR, sHRz, and “participant” as predictor variables. The model predictions are plotted against the measured RPE values in Figure 2. All predictors were significant (each *p* < 0.001). As indicated by the conditional *R*^2^, the predictors explained 45% of the RPE variance together (95% CI: 0.18). As indicated by the marginal *R*^2^, cHR, and sHRz explained 27% of the variance together (95% CI: 0.19). The significance of the fixed predictors confirms a two-component structure of RPE which has been previously reported on adults in the literature. The significance of the random predictor confirms that the relationships between RPE and heart rate data differed between the participants. Therefore, semi-partial correlation coefficients for cHR and sHRz were calculated for each participant by individual regression analyses. The analyses yielded median semi-partial correlation coefficients of *r*_s _= 0.43 (interquartile ranges—IQR: 0.59, mean: 0.37, 95% CI: 0.27) for cHR and *r_s_*_ _= 0.45 (IQR: 0.24, mean: 0.43, 95% CI: 0.18) for sHRz. The correlation coefficients were transformed with Fisher's z-transformation for the following analyses.

A confirmatory factor analysis was performed to test the hypothesis that the results from the cognitive performance tests and the BRIEF questionnaire are related to the variance component of RPE exclusively explained by cHR. The cognitive measures and the z-transformed correlation coefficient of cHR were included in the analysis. Perseveration was not included due to violations of the assumption of normality.

The *χ*^2^-Test of the factor model was not significant [*χ*^2^_(2) _= 1.87, *p* = 0.392]; thus, the global null hypothesis assuming the described relationship was not rejected. The descriptive measures were in the ranges defined *a priori* (CFI = 1.00, TLI =1.03, RMSEA ≤0.01, SRMR = 0.05). Moreover, the factor loadings of all variables were significant (z-transformed *r*_s_ of cHR: *p* = 0.039, z-standardized factor loading: 2.06; Bit/s: *p* = 0.002, z-standardized factor loading: 3.14; unique designs: *p* = 0.006, z-standardized factor loading: 2.76; BRIEF: *p* < 0.001, z-standardized factor loading: 3.57). Introducing the z-transformed correlation coefficient of sHRz to the factor model yielded an insignificant factor loading for this variable (*p* = 0.552, z-standardized factor loading: −0.60).

### 3.4. Production of intensities in training session 2

**Figure 3:**
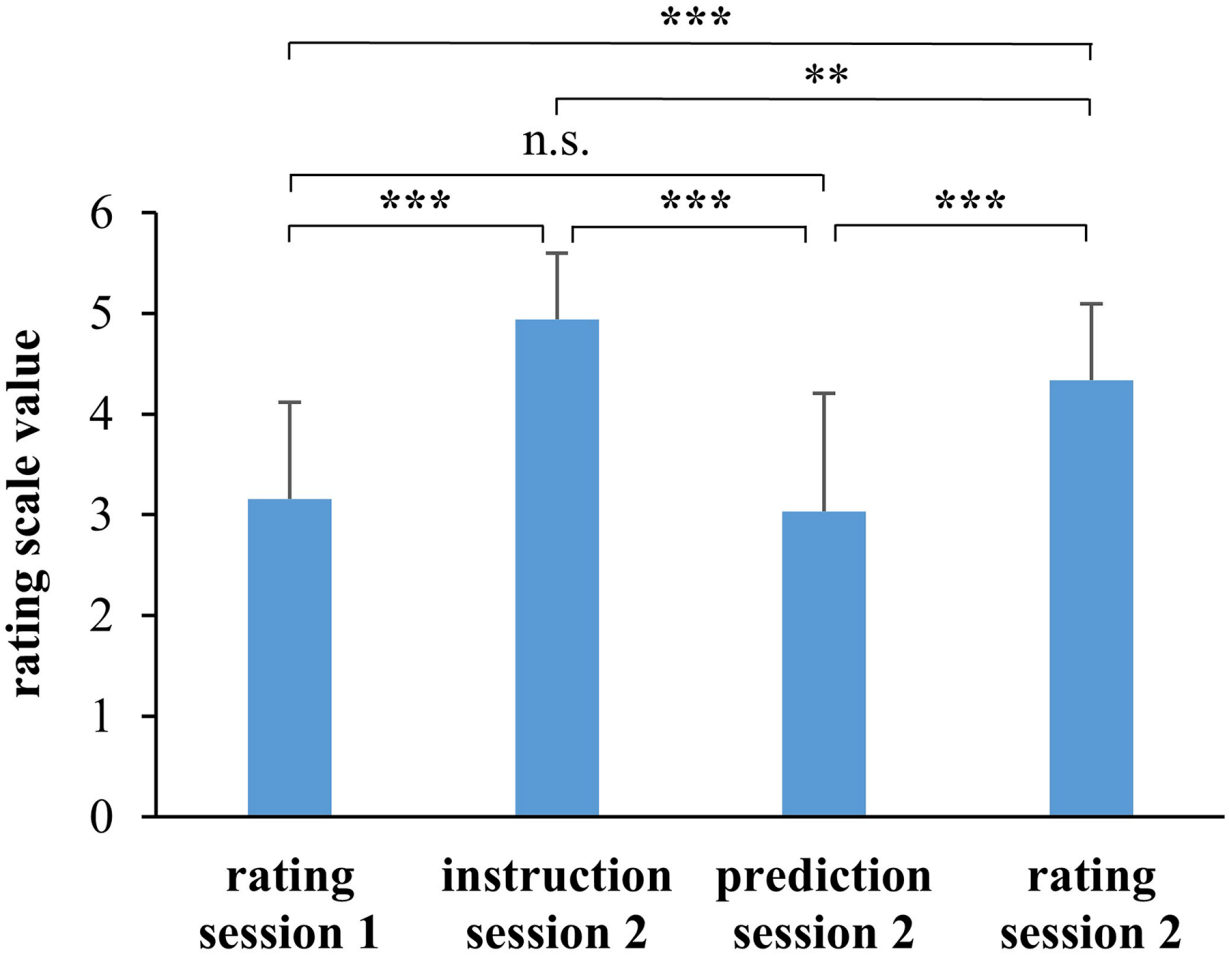
Mean scale values for the instructed, predicted, and rated intensities in training session 2 and rated intensity in training session 1. n.s., not significant. *p < 0.05, **p < 0.01, ***p < 0.001.

In the second training session, the participants performed the same training tasks as in session 1, but now received instructions concerning the production of training intensities. The instructions were on average 1.76 (SD: 0.49, 95% CI: 0.40) rating scale values higher than in the first training session. Figure 3 contrasts the subjective ratings from training session 1 and the rated, instructed as well as predicted RPE in training session 2. The prediction for RPE was calculated for each participant with his regression equation from training session 1 describing the relationship between RPE, cHR, and sHRz. Inserting the cHR- and sHRz-data from training session 2 into the regression equations yielded predictions for RPE in training session 2. An ANOVA revealed significant differences between the four variables [*F*(3.69) = 62.22, *p* < 0.001, *ɳ*_p_^2 ^= 0.73]. Post-hoc tests confirmed that the participants rated effort significantly higher in training session 2 than in session 1 (*d* = 1.22, *p* < 0.001) and higher than predicted from the heart rate data (*d* = 1.19, *p* < 0.001).

**Figure 4:**
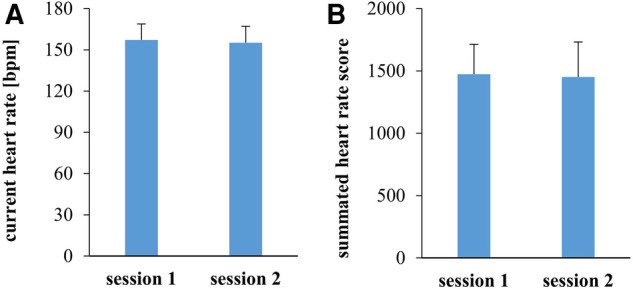
Current heart rate at the time when the perception of effort was rated (**A**) and the summated heart rate zone score (**B**) in training sessions 1 and 2. Data are illustrated as between-subject means and standard deviations.

The RPE prediction for session 2 did not differ significantly from RPE in training session 1 (*d* = −0.19, *p* = 0.780). Accordingly, neither cHR nor sHRz differed significantly between both sessions (Figure 4, cHR: *F*(1.24) = 1.87, *p* = 0.184, *ɳ*_p_^2 ^= 0.07; sHRz: *F*(1.24) = 0.28, *p* = 0.605, *ɳ*_p_^2 ^= 0.01).

### 3.5. Effects of age on estimation and production performance

**Figure 5:**
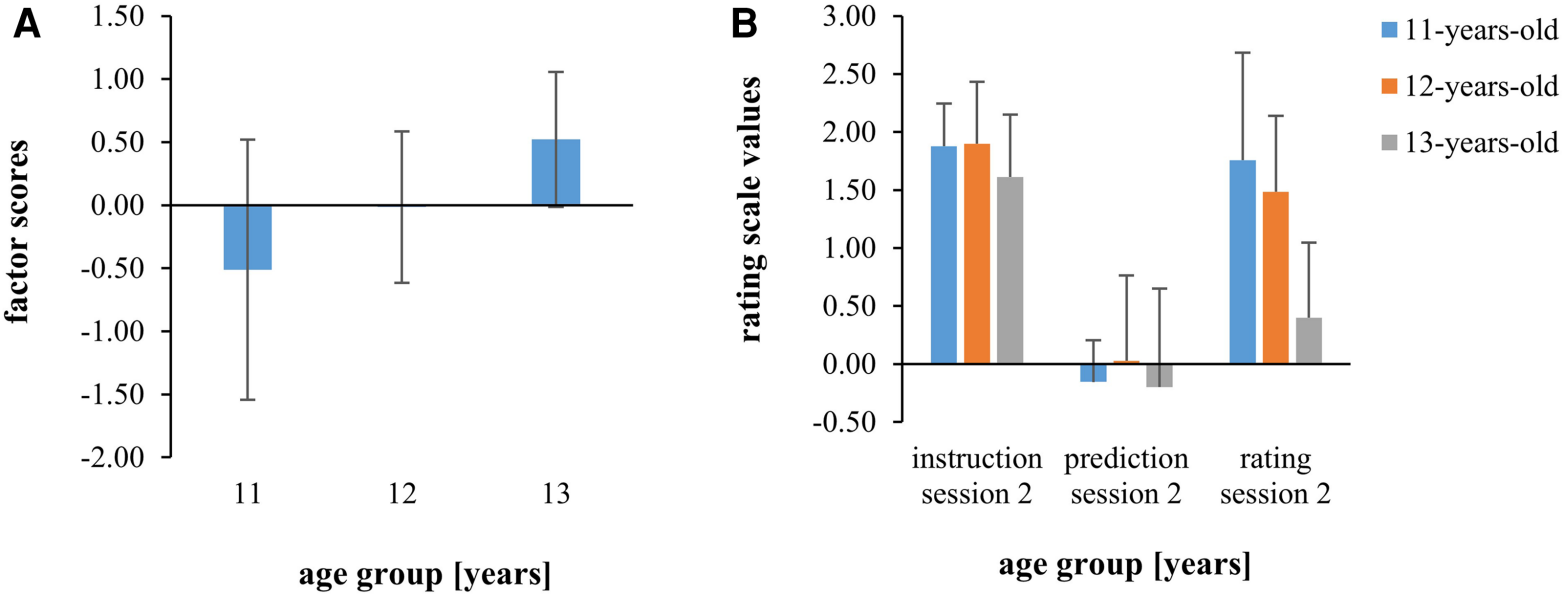
Scores from the factor analysis differentiated by age groups (**A**) mean scale values for the instructed, predicted, and rated intensities in training session 2 normalized by subtraction on rating scale values from training session 1 differentiated by age group (**B**) shown are between-subject means and standard deviations.

If the competencies for estimating and producing intensities are related to maturation, it is expected that older children have higher estimation- and production competencies than younger children. To investigate the influence of age on the estimation competence, the individual factor scores from the factor analysis in Section 3.3 were compared across the three age groups of the present study. As shown by Figure 5A and statistically confirmed by ANOVA, the factor scores increase significantly with age [*F*(2.22) = 4.07, *p* = 0.031, *ɳ*_p_^2 ^= 0.27]. In the post-hoc analysis, thirteen-year-old children had significantly higher factor scores than eleven-year-old children (*d* = 1.26, *p* = 0.024). Twelve-year-old children did not differ significantly from eleven-year-old (*d* = 0.57, *p* = 0.421) or thirteen-year-old-children (*d* = −0.95, *p* = 0.363).

Effects of age regarding the production performance in training session 2 were analyzed by comparing the instructed, the predicted, and the rated effort of session 2 between age groups. The data were normalized by subtracting the ratings from session 1. This normalization was necessary to meet the homogeneity assumption, which is violated when considering the non-normalized data. The results are illustrated in Figure 5B. The ANOVA yielded a significant effect of age [*F*(2.21) = 4.08, *p* = 0.032, *ɳ*_p_^2 ^= 0.28] as well as a significant interaction measure × age [*F*(4.42) = 3.50, *p* = 0.015, *ɳ*_p_^2 ^= 0.25]. Post-hoc comparisons confirmed significantly lower RPE 2-values of the thirteen-year-old than the eleven-year-old children (*d* = −1.69, *p* = 0.027). Other group differences were not significant.

## 4. Discussion

The present study investigated children's competencies to rate perceived effort and produce predetermined intensities during soccer training. A review by Groslambert and Mahon (10) concluded that children aged 8–12 years can differentiate only four intensities. Recently, Kasai et al. (23) concluded that children are capable of assessing effort in a more fine-grained manner, provided that a child-appropriate effort scale (e.g., the OMNI-scale) is used and the examination is conducted in a controlled laboratory setting with an incremental exercise protocol. Such procedures might simplify the rating of perceived effort, because the load increases proportionally with the test duration, making future loads predictable. The present study investigated children during soccer training, in which they cannot base their ratings on the expectation of increasing load profiles, because intensities vary over time (the load profiles over time can be assessed from the Data Sheet). The hierarchical linear regression analysis on the data of the first training session shows high associations between the subjective and the objective load measures (Section 3.3). From these results, it can be concluded, that children aged 11–13 years are able to rate perceived effort during soccer training.

Two heart rate measures explain independent portions of the RPE variance. cHR represents a punctual measure and reflects the current physiological strain. sHRz is based on the summation of all heart rate measures during a training task and thus reflects the cumulative training load of this task. This differential result might contribute to the elucidation of a conceptual discourse that reveals itself in terms of terminology. It is highly debated whether “rating of perceived effort” or “rating of perceived exertion” denote the same or different phenomena and whether one term is more adequate than the other in describing the subjective perception during exercising. According to Abbiss et al. (13), effort refers to “the amount of … energy given to a task” and exertion to “the strain experienced during a physical task”. Following this view, the RPE component explained by cHR might characterize what is described by the term effort, and the RPE component explained by sHRz might reflect exertion.

Although the results of training session 1 show that children of this age can already make reasonable statements about their perceived effort and exertion, the mean correlation coefficient between cHR and RPE (0.43) was lower than in studies with adult soccer players [0.71 and 0.61 (12, 15)]. In contrast, the mean correlation coefficient between sHRz and RPE (0.45) was comparable to those of adults [0.42 (15)]. The previously reported differences between children and adults in RPE competence (10, 31) might thus be attributed to the variance component of RPE that is explained by the current heart rate. In accordance with the above reasoning, this component is likely related to the perception of effort. Age was also a significant factor in the present study. The thirteen-year-old children achieved higher factor scores in the factor analysis than the eleven-year-old children. This shows that the competence subsumed in the factor is more pronounced in thirteen-year-olds than in eleven-year-olds. The lower divergence between measured and predicted RPE in thirteen- compared to eleven-year-old children in training session 2 also reflects better estimation performance in the older age group.

The results of the first training session also point to inter-individual differences regarding the RPE-competence. It is assumed that the RPE-competence is related to cognitive abilities that develop during childhood and adolescence. The result of the confirmatory factor analysis supports this view and the more specific hypothesis of the present study because it shows that the performances in the number connection test, the five-point test as well as the results from the BRIEF questionnaire are related to the RPE component exclusively explained by cHR (12, 15, 34). Noteworthy is the outcome of the BRIEF questionnaire. Higher RPE competencies, faster processing speed, and spontaneous flexibility were associated with higher scores in the behavioral regulation index. A similar result has been reported by Schmitz and Sommer (15) for adult soccer players. Higher scores indicate that these participants feel less inhibitory and emotional control, less flexibility, and lower performance regarding the monitoring of their actions in their daily life. Since the questionnaire captures the own view of participants, these results might indicate that participants with higher performance regarding RPE and information processing have a more critical view on their executive control.

The second part of the study investigated whether children are able to produce instructed intensities during soccer training. A second training session was performed similar to the first session, with the difference that the participants were instructed to reach predefined intensities. Since the participants had rated effort comparably low in the first session, it was necessary to instruct on average higher intensities in the second training session. The children indeed rated effort significantly higher in the second than in the first session (Figure 4). However, none of the heart rate measures reflect an intensity increase. Thus, the children perceived a higher effort, but this effort was not reflected by the measured physiological parameters. The effect was somewhat less pronounced in the 13-year-olds, although no changes in heart rate parameters were measurable in them either. In a study from Eston and Lamb (46), children realized lower intensities when instructed to produce scores of 3, 5, and 7 on the RPE scale compared with a previous situation in which they self-rated intensities as 3, 5, and 7 on the RPE scale. The same might have happened in the present study, with a higher RPE in session 2 resulting in nearly similar heart rates compared to session 1.

A systematic literature review suggested that children perform worse in the production compared to the estimation paradigm. However, only a few studies directly compared estimation and production performance (23, 31). Lamb (5) argued that they might not be compared as the underlying competences differ too much. Other criticisms concerned differences between tasks (one often continuous and the other intermitted) or the statistical analyses (5, 31). In the present study, the exercise protocols of training session 1 and 2 were similar, but different statistical analyses had to be performed. Therefore, comparisons can only be made on an argumentative level. The results indicate that estimation and production performance diverged in the present study. Though the children were competent in rating perceived effort, they failed to produce instructed intensities during the second soccer training. This might indicate that the production of intensities requires further competencies beyond the RPE competence which are not yet sufficiently developed at this age. As will be discussed in the following, the production competence appears to depend on intrinsic factors. Moreover, production performance may also depend on environmental constraints like the presence of other individuals.

The competence for regulating exercise intensity seems dependent on age. Menting et al. (47) reported that children aged 10–14 years regulate their running speed during an endurance task in a goal-directed way, whereas younger children apply all-out pacing, which means that they start very fast and then decrease their running speed over time. The behavior of older children still differs from that of adults. Another moderating factor for intensity regulation might be cognitive development. By controlling for age, Micklewright et al. (48) showed that children with more advanced cognitive development realize a more adapted pacing strategy, i.e., a more adequate intensity regulation, than children with lower cognitive development. According to Holgado and Sanabria (49) as well as Sakalidis et al. (50), executive functions might again play an essential role. Since estimation and production performance sometimes differ, further executive functions may be relevant for the regulation of intensities. From a developmental perspective, working memory, action monitoring, affective decision-making, and goal setting might be candidates as they are not fully developed until adulthood (51, 52). As argued by Hyland-Moks et al. (53) as well as Sakalidis et al. (50), it is plausible to assume that these functions are involved in the regulation of exercise intensities. Another explanatory approach is the probable influence of environmental factors, which seem to change with age. Performing sports together with other people seems to negatively affect intensity regulation in children but positively affect intensity regulation in adults (47). A similar effect has been found in individuals with intellectual disabilities who have less developed executive functions compared to individuals without intellectual disabilities (54). Since soccer is a team sport, these findings may explain why children in the present study were not able to change their intensities in the second training session.

Several limitations of the present study need to be addressed. Edward's heart rate zone method requires determination of HRmax. Since the testing effort for each child in the present study was already high, the measurement of HRmax was omitted. Instead, HRmax was approximated with a formula, which is recommended in cases when HRmax cannot be measured with appropriate tests (23). Nevertheless, measuring HRmax is recommended for future studies, for example, with a 20 m shuttle run test (6), which is a widely used test in soccer.

As an alternative to the measure used in the present study, other measures of cumulative exercise intensity might be considered in both research and practical applications. Although several studies reported large correlations between sHRz and RPE in children and adolescents (17–20, 22, 55), it can be questioned whether the heart rate zones should have the same thresholds for all age groups. To individualize thresholds, other physiological parameters besides HRmax might be used. For example, Lucia et al. defined three heart rate zones in relation to the ventilatory thresholds (56). Impellizzeri et al. (55) found larger correlations between RPE and Lucia's zone score than between RPE and Edward's zone score in adolescent soccer players (*r* = 0.70 vs. *r* = 0.64). Nonetheless, sHRz is useful when no other physiological parameters can be collected, which is usually the case in amateur sports.

It is shown that the RPE-competence is related to the cognitive development of the participants. Thus, RPE-scales should be individually validated by setting RPE in relation to heart rate measures. The present study results suggest that cHR as well as sHRz should be determined, as they independently explain variance in RPE-competence. The children of the present study were able to use the modified OMNI scale to rate perceived effort in a field situation. A study from Eston et al. (57) suggests that children's perceived effort increases exponentially with linearly increasing load. Therefore, a curvilinear rating scale such as the Eston-Parfitt (E-P) scale might be more appropriate in the work with children. Furthermore, as repeated use of the scales increases the reliability of the measurements, adequate familiarization phases should be realized when new scales are introduced (8, 26). Finally, coaches must be aware of the responsibility in dealing with intensity instructions. First, intensity instructions might have an impact on children's perceived effort. Second, as long as it is not clear whether children can produce prescribed intensities in complex sports situations, coaches might carefully reflect on whether to provide intensity instructions during a given task or not. Which factors prevent or affect the production of intensities should be investigated in further studies.

## 5. Conclusion

The results from the present study show that children aged 11–13 are able to rate perceived effort during soccer training. Correlations of two different heart rate-based measures with the rating score confirm an assumed multi-component structure of RPE. The competence to rate perceived effort seems to be less developed in younger compared to older children and compared to adults. It was confirmed that the rating performance is related to the performance in cognitive tests supporting the hypothesis that RPE competencies depend on cognitive development. In a second training session, the children were unable to change their intensities in response to instructions. Future research needs to identify the underlying factors of this competence.

## Data Availability

The original contributions presented in the study are included in the article/**Supplementary Material**, further inquiries can be directed to the corresponding author.
