# Supplementary figures and images for: Children can rate perceived effort but do not follow intensity instructions during soccer training
Source: Front Sports Act Living. 2023 Nov 2;5:1251585. doi: 10.3389/fspor.2023.1251585 (PMC10652393; doi:10.3389/fspor.2023.1251585)

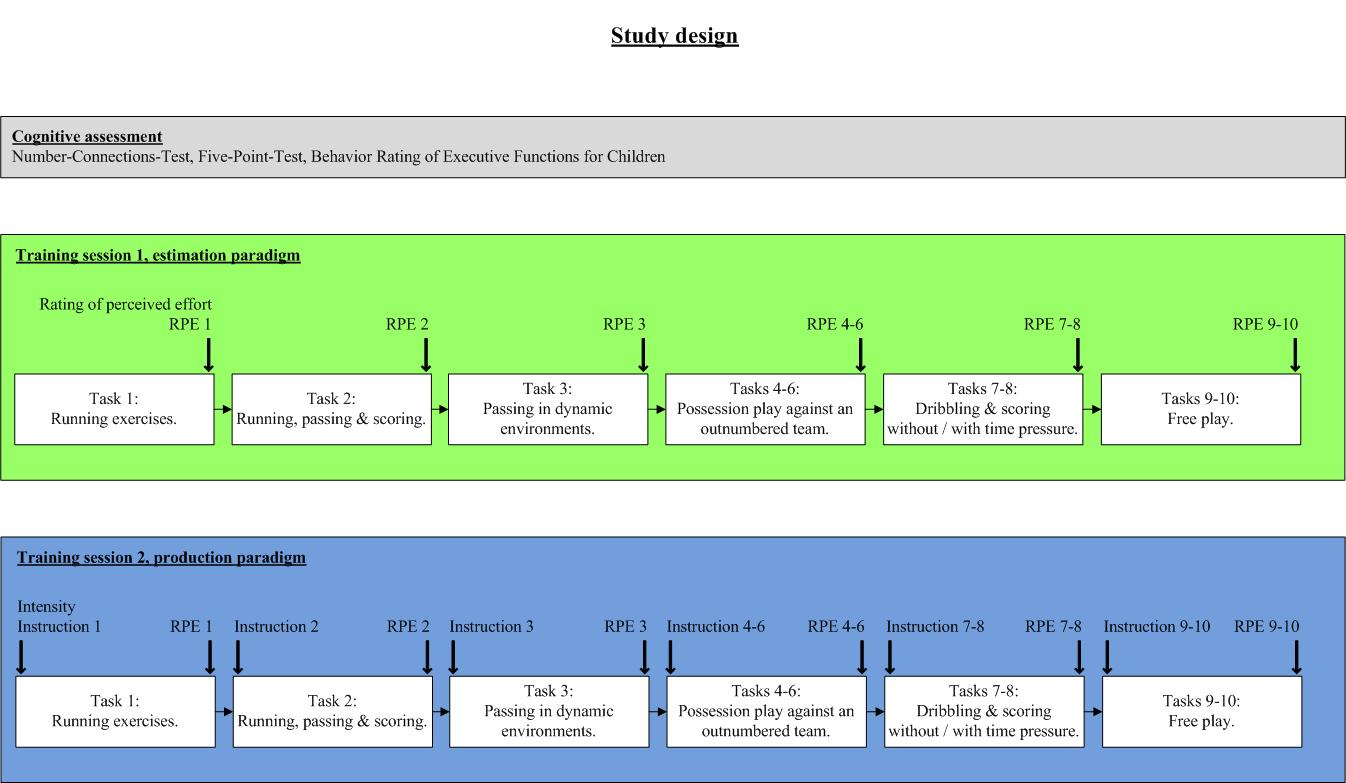

Supplement: Supplementary file 2 [file Image1.jpeg]
